# Evolutionary Overview of Land Consolidation Based on Bibliometric Analysis in Web of Science from 2000 to 2020

**DOI:** 10.3390/ijerph19063218

**Published:** 2022-03-09

**Authors:** Xin Xu, Qianru Chen, Zhenhong Zhu

**Affiliations:** Institute of Ecological Civilization, Jiangxi University of Finance and Economics, Nanchang 330013, China; 2202021937@stu.jxufe.edu.cn (X.X.); 2202010105@stu.jxufe.edu.cn (Z.Z.)

**Keywords:** Bibliometrix, land consolidation, thematic evolution, reclamation, Biblioshiny

## Abstract

Land consolidation is widely used as a powerful tool for land use management in many countries. In order to objectively reveal the current research status in the field of land consolidation, this paper uses the Bibliometrix and Biblioshiny software packages, and VOSviewer to analyze the literature in the field of land consolidation in the last 20 years of the Web of Science Core Collection Database. The results show that: (1) In the past two decades, the annual publication of papers on land consolidation rose. It can be divided into three stages: 2000–2007 for the embryonic period, 2008–2012 for the long-term, and 2013–2020 for the high-yield period. (2) Land consolidation studies covered 68 countries or regions. The top three countries were China, Poland, and the United States. China and the United States played an important role in international cooperation in the field of land consolidation, and Turkey mainly conducted independent research in the field of land consolidation. (3) Land consolidation, reclamation, China, remote sensing, and land fragmentation were the high-frequency keywords in the field of land consolidation in recent years. (4) The research focusing on the field of land consolidation involved its development course, its impact on ecosystem services, and the evaluation of its benefits. (5) The theme of land consolidation studies was shunted and evolved over time, and nine evolution paths could be summarized in the studies of cultivated land fragmentation, development course of land consolidation, and impacts of land consolidation on soil. Finally, this paper predicted the future research directions of land consolidation: exploring new methods for evaluating the benefits of land consolidation, the scale effects of the impact of land consolidation on ecosystem services, research on the mechanism and comprehensive effects of land consolidation on soil, research on land consolidation and rural revitalization, and land consolidation theory research.

## 1. Introduction

Land is the basic resource for human survival and development [1,2]. The rapid development of the social economy and population growth increase the demand for land. However, the limited land cannot provide more space and resources for human beings, and the contradiction between man and land becomes prominent [2,3]. Currently, more than half (54%) of the world’s population lives in urban areas, and this proportion is expected to reach 66% by 2050 [4]. Food production is expected to increase by 70% (by 2050) to satisfy the growing population, and land requires a more sustainable management mode [5]. It demonstrates the need for the rational and efficient use of the world’s limited land resources in order to maximize the provision of products and services to the growing food needs of humankind.

Land consolidation refers to the activities of integrated management of unused, inefficient and idle, damaged, and degraded land to meet the needs of human production, life, and ecological functions. It is the general term for land development, consolidation, reclamation, and restoration [6]. The function of land consolidation is reflected in many ways. As an effective tool to supplement cultivated land, land consolidation plays an active role in achieving the balance of arable land [7], ensuring national food security [8], and promoting rural revitalization [9]. It has been widely used all over the world in recent decades. Land consolidation policies have gradually shifted from initial agricultural production objectives to a means of supporting rural development [10]. Moreover, land consolidation has far-reaching effects on promoting the scale of agricultural production and improving the competitiveness of agricultural products, adjusting the structure of land use, developing modern agriculture, alleviating ecological risks, and improving agricultural production efficiency [11,12,13,14,15].

Bibliometrics is a quantitative analysis method that uses mathematical and statistical tools to measure the interrelationship and impact of publications in a particular research area [16]. It enables researchers to sketch out complex knowledge maps that represent the structure of knowledge in a field of study and study their properties through statistical and mathematical methods [17,18,19]. As a powerful tool for analyzing the field of knowledge and revealing its cognitive–epistemological structure [20], it provides a macro-overview of a large number of academic studies and reliably identifies influential research, authors, journals, organizations, and countries [21].

Scholars have used large-scale data sets and bibliometric methods to carry out bibliometric analysis in the field of land consolidation, and obtained valuable research results. However, most of the research focuses on keyword co-occurrence analysis, journal sources, and author publications, while studies on the context of historical citation, high-frequency keyword clustering analysis, subject evolution, future development direction prediction, etc. are rare. Therefore, the Bibliometrix and Biblioshiny software packages in the *R* tool are adopted in this paper to systematically measure the literature of land consolidation in the Web of Science core collection database set during 2000–2020. This study aims to solve the following scientific questions.

(1) How are the keywords in the field of land consolidation clustered?
(2) What countries have cooperated in the field of land consolidation?
(3) How did the history of citations in the field of land consolidation develop?
(4) What is the focus and direction of future research in the field of land consolidation?

## 2. Data Sources and Research Methods

### 2.1. Data Sources

Web of Science is the world’s largest and most subject-covering comprehensive academic information resources, including more than 8700 core academic journals in various fields of natural sciences, engineering, biomedicine, social sciences, and arts and humanities. This paper took the core collection in the Web of Science database as the data source. The search term included TI = “land consolidation” or TI = “land reclamation”. The document type was limited to “Article”, and the retrieval time was 2000–2020. The languages were English and Chinese. After pre-processing, such as de-duplication and the removal of irrelevant data, a total of 599 papers in the field of land consolidation were obtained. Among them, 594 papers were in English, accounting for 99.17% of the total number of articles. The downloaded data were saved in a text format.

### 2.2. Research Methods

Bibliometric analysis provides a comprehensive overview of a large body of research literature and further developed previously unevaluated insights by allowing quantitative and objective identification of past and present research topics [22].

The Bibliometrix *R* package provides a set of tools for quantitative research in scientific metrology. It is written in *R*, an open-source environment and ecosystem. The existence of a large number of effective statistical algorithms, access to high-quality numerical routines, and integrated data visualization tools may be the strongest qualities of *R* languages in scientific computing over other languages [23].

Biblioshiny was developed by Massimo Aria in a secondary development of the Bibliometrix-based Shiny package in the *R* language, encapsulating Bibliometrix’s core code and creating a web-based online data analysis framework. Users can carry out relevant scientific measurement and visual analysis work on the interactive web interface, which reduces the user’s threshold of use and the intensity of information input, to a certain extent [24].

**Figure 1:**
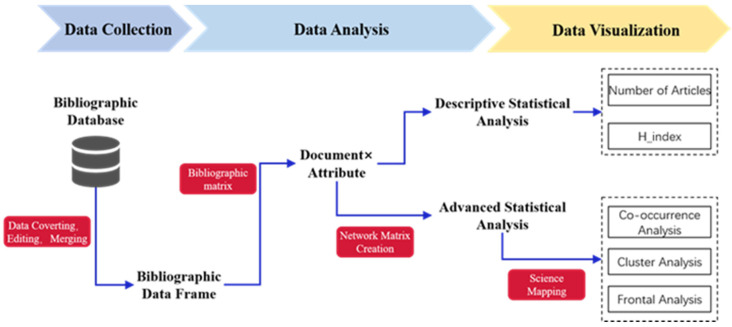
Bibliometrix and the recommended science-mapping workflow.

Developed by Nis Jan van Eck and Ludo Waltman, VOSviewer is also widely used in bibliometric analysis due to its more aesthetically pleasing visualization processing, in particular, keyword co-occurrence analysis [25,26]. The specific Bibliometrix and science-mapping workflow is shown in Figure 1.

## 3. Results Analysis

### 3.1. Distribution of Annual Documents

**Figure 2:**
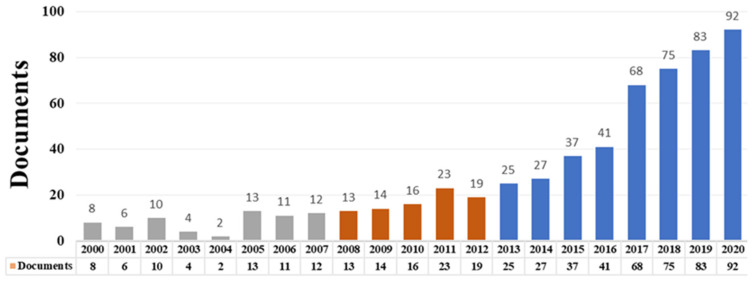
Number of land consolidation research documents published from 2000 to 2020.

The analysis of the distribution of literation volume from the time series can reflect the trend of study [24]. Figure 2 shows that, from 2000 to 2020, the number of research studies published in the field of land consolidation fluctuated slightly, but is on an upward trend overall. Combined with the macro-policy changes in land consolidation, it was divided into three research stages: 2000–2007, 2008–2012, 2013–2020. The 2000–2007 period was the germination period of land consolidation research. The annual publication volume was very small with few differences. The 2008–2012 period was the long term. Although the growth rate of literature publication was not obvious, but compared with the previous stage, the annual publication volume was more stable, indicating that the function of land consolidation attracted the attention of scholars. The 2013–2020 period was the high-yield period with an obvious growth rate. The number of publications reached a peak of 92 in 2020. Combined with China’s comprehensive land consolidation policy formulated in 2013, it shows that the research on land consolidation in recent years is of important practical significance.

### 3.2. Analysis of Cited Papers in Land Consolidation Research

#### 3.2.1. Annual Development Trend of Citations

**Figure 3:**
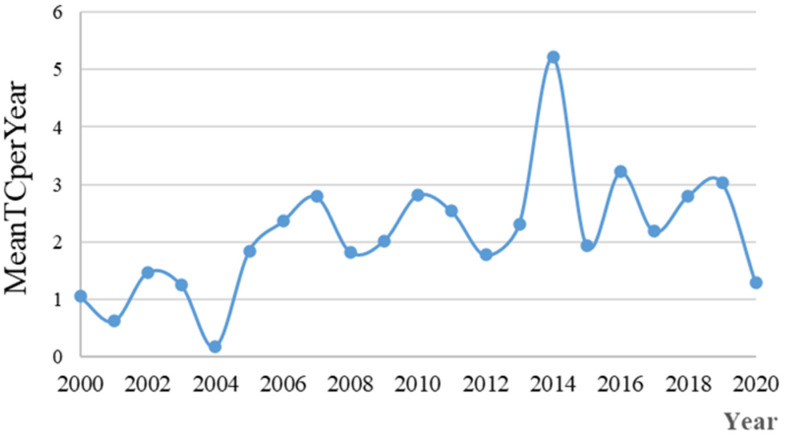
The number of annual cited papers.

As can be seen from the average citation distribution of papers per year (Figure 3), the citations were low in 2000–2004. Especially in 2004, the citation was lowest, showing that the research period was in its infancy. The cited frequency was at an average of 3.0 in 2006–2012 and 2014–2016. It reached its highest level in 2014 at 5.18. At the same time, the fluctuation was greater than the annual change in paper yield. In 2018–2020, the average citation of papers on land consolidation was in a declining stage, while the volume of published papers was on a steady upward trend. This indicates that, despite the various research directions on land consolidation, the influence of papers was decreasing.

The highest yield of cited papers was in 2014 at 5.20. From 2012 to 2014, the average citation of highly cited papers increased explosively. Most scholars in this period mainly analyzed the effects of land consolidation based on multi-type land policies. For example, Li analyzed the negative impacts of land consolidation on China’s coastal ecosystem and its services, and called on China to strengthen the construction of laws and regulations, improve marine spatial planning, and fully assess the negative impacts of land consolidation [27].

#### 3.2.2. Historical Cited Papers of Land Consolidation Research

Using the historical citation visual analysis in the Bibliometrix installation package in *R* Studio, 20 nodes were selected, and the pioneering works and some classic studies in this field were found. CiteScore is the youngest indicator, which was released in December 2016 [28]. In this paper, LCS and GCS indicators were used to analyze the research methods and contents of classical literature. LCS refers to the reference score in the downloaded paper dataset, and GSC refers to the reference score in the Web of Science core collection database.

**Figure 4:**
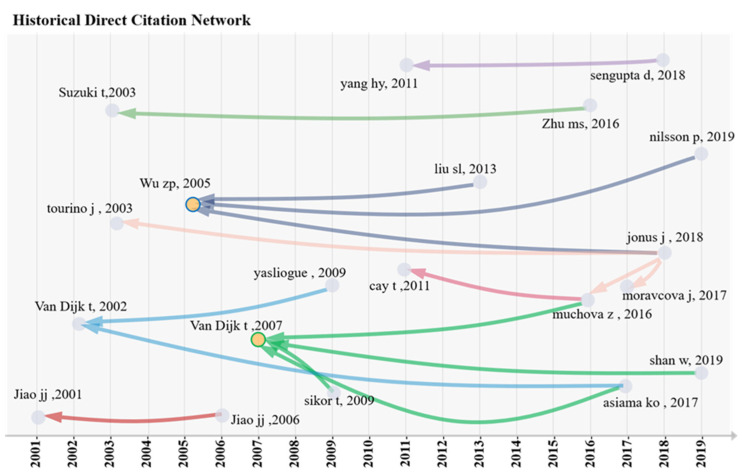
Historical direct citation network of top-cited papers in the field of land consolidation during 2000–2020.

As can be seen from Figure 4, the earliest node in the literature on land consolidation was an article published in *Ground Water* in 2001 entitled “*Analytical Studies on The Impact of Land Reclamation on The Water Flow*”. On the basis of the negative impacts that land reclamation will have on the ecosystem and its services, this paper analyzed the influence of land reclamation on groundwater flow. The results showed that the larger the scale of land reclamation, the more significant the increase in the horizontal line of the coast [29].

Several classic articles emerged between 2005 and 2007 (Figure 4 clearly cites the relationship). Terry’s article in 2007 in *Geoforum* entitled “*Complications for Traditional Land Consolidation in Central Europe*” had four distinct chains of citations, and its citation frequency of 39 was also the highest of the LCS. This article described the complexity of implementing traditional land consolidation in Central Europe from factors such as unfavorable macro conditions, absentee landowners, land ties, and unfinished privatization [30]. Terry first published a paper on land consolidation in 2002 in *European Planning Research*, entitled “*Export of Planning Knowledge Needs Comparative Analysis: The Case of Applying Western Land Consolidation Experience in Central*”. Its innovation lies in putting forward cross-border knowledge transfer research comparative analysis by demonstrating land consolidation as a case [31].

Wu’s article in *China Economic Review* in 2005 entitled “*Land Consolidation and Productivity in Chinese Household Crop Production*” also presented three significant chains of citation relationships. As shown in Table 1, both LCS and GCS ranked second, which were 17 and 81 respectively. This indicates that the literature is not only a classic document in the field of land consolidation, but also favored by scholars in many fields with a strong cross-cutting with other disciplines. Based on raw data from 227 Chinese households, this paper evaluated the effectiveness of land consolidation projects in the agricultural comprehensive development plan. The results showed that the land consolidation project improved the land quality and contributed 1.52% to the crop yield [32]. The LCS and GCS of Hoeksema’s article in *Irrigation and Drainage* in 2007 entitled “*Three Stages in the History of Land Reclamation in the Netherlands*” are at a high level. This paper briefly introduced the three stages of land reclamation in the history of the Netherlands and laid a foundation for other scholars’ research [33].

Yang et al. [34] published an article in *Bird Conservation International* in 2011 entitled “Impacts of Tidal Land Reclamation in Bohai Bay, China: Ongoing Losses of Critical Yellow Sea Waterbird Staging and Wintering Sites”. As shown in Table 2, the GCS is as high as 90, while the LCS is not ideal, which is 6, and only one lead chain appeared. Based on remote sensing technology to collect data, this paper analyzed the influence of wetland reclamation on water birds on the Bohai coast of China.

### 3.3. Analysis of Main Researchers

**Figure 5:**
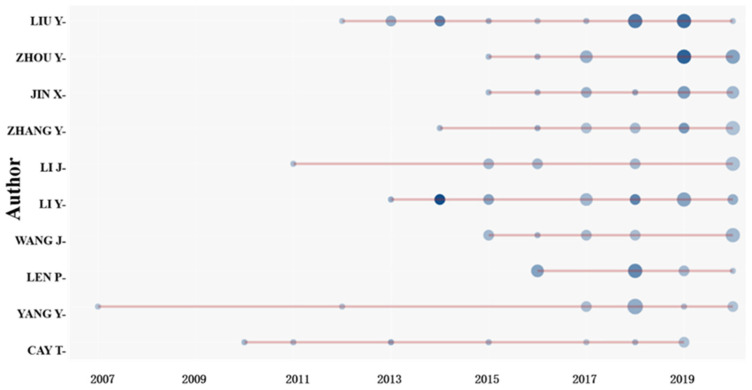
Authors’ production over time in the field of land consolidation.

A total of 1585 authors were involved in the paper data set on land consolidation, of whom Liu, Zhou, and Jin were the top three authors, with 12, 12, and 11 publications, respectively (Table 3). In this field, the largest number of publications was from China’s Liu, with a h-index of 7, g-index of 12, and total number of citations of 349. It indicates the high quality and great influence of Liu’s papers. As can be seen from Figure 5, Liu’s most frequently cited paper appeared in 2019 (the darkest color of the graph), which was cited 28 times. Liu’s article entitled *“Land consolidation boosting poverty alleviation in China: Theory and practice”*, published in *Land Use Policy* in 2019, analyzed the mechanisms and dynamics of land consolidation to alleviate poverty in China. The results showed that land consolidation plays an active role in increasing the cultivated land area, promoting the agricultural production scale, improving the conditions of rural production, and reducing ecological risks [35].

Zhou’s most frequently cited paper also appeared in 2019. His article entitled “*Land consolidation boosting poverty alleviation in China: Theory and practice*” revealed the link between land consolidation and poverty alleviation [35]. The total number of citations of Li from China was the highest (Table 3), which was 455, and his most frequently cited year was 2014.

Hirsch believes that the h-index is not only an acceptable tool for measuring the importance, significance, and broad impact of authors’ cumulative research contributions, but also for assessing current paper volumes and predicting authors’ future performance, as this indicator combines productivity and impact [36,37]. However, comparison of the h-index alone may be misleading due to the loss of citation information [38]. To compare the influence of authors in this field at different times, the m-index can be introduced. To be specific, m = h/n, where n indicates the age of the author’s publication in the field [28].

Len’s m-index was the highest, which was 1.167. Len has been publishing papers in the field of land consolidation since 2016, while the most frequently cited year was 2018. For example, Len’s article in *Computers and Electronics in Agriculture* in 2018 entitled “*An algorithm for selecting groups of factors for prioritization of land consolidation in rural areas*” presented a general algorithm for factor group selection. Based on similar areas of two regions, but varied significantly in spatial structure in Poland, this paper proved that the algorithm could prioritize land consolidation activities in these areas [39].

### 3.4. Analysis of Distribution Characteristics of Major Research Countries/Regions

The distribution characteristics of major research countries/regions reflect each country’s influence in the field of land consolidation and provide conditions for further exploitation on the reasons for different degrees of influence. The dataset used in this article was published in 68 countries or regions. The top 25 published papers were distributed as follows: nine Asian countries (China, Turkey, Korea, Japan, India, Singapore, Iran, Malaysia, and Thailand), three American countries (the United States, Canada, and Brazil), two Oceania countries (Poland and Australia), and eleven European countries (Netherlands, United Kingdom, Spain, Czech Republic, Italy, Russia, Germany, France, Serbia, Slovakia, and Romania).

**Figure 6:**
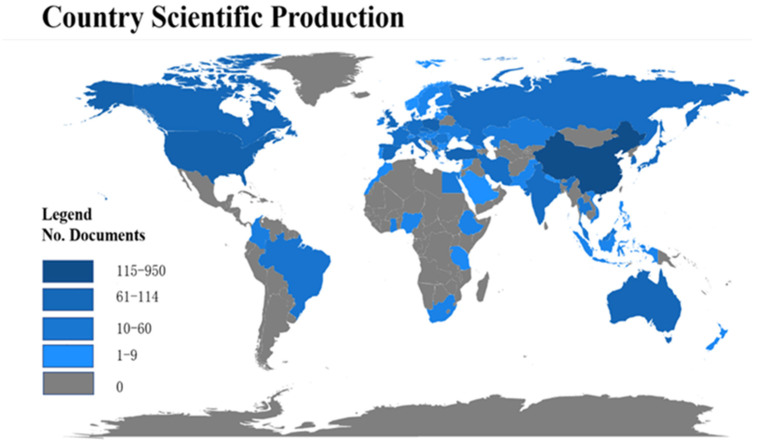
Scientific production distribution in the field of land consolidation.

**Figure 7:**
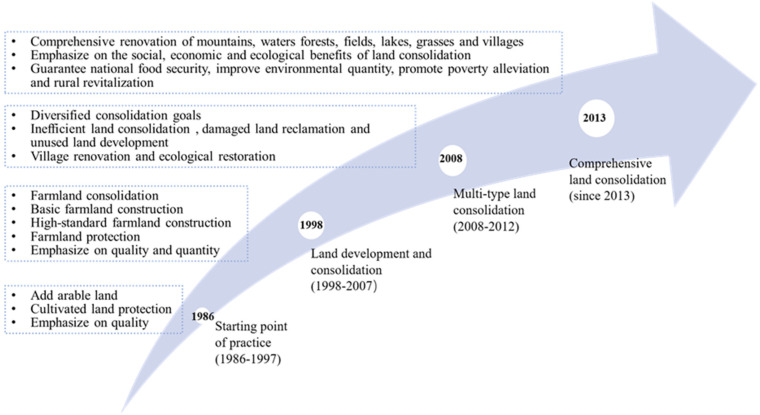
The history of land consolidation in China. Source: Land consolidation and rural revitalization in China: Mechanisms and paths [43].

As can be seen from Figure 6, papers on land consolidation were published mainly in Asia and Europe. Specifically, China is the only developing country in the top three, with several times the number of studies of that of other countries, accounting for about 50% of the total production. The reasons for such high production may be China’s far-reaching history in land consolidation and the evolution of related policies (Figure 7). In 2003, the Chinese “National Land Development and Consolidation Plan” specifically elaborated the objectives, principles, key areas, etc. of land consolidation, providing a guiding role for the nationwide implementation of land consolidation. Before 2008, the prevailing idea of “emphasized quantity over quality” in land consolidation increased the cultivated land area in China [40]. However, grain production at this stage still remains stagnant. In 2011, the Ministry of Natural Resources issued the “*High-standard Basic Farmland Construction Specification*”, aiming at improving the quality and productivity of arable land. It meant that the quality of arable land was as important as quantity. In March 2012, the Chinese “National Land Consolidation Plan (2011–2015)” was implemented [41]. It meant the beginning of comprehensive land consolidation in China, which included agricultural land, construction land, and unused land, and more attention was paid to the protection of the ecological environment. However, the average citation for each paper was 13.44. It was lower than that of the United States of 17.90, while its paper volume ranked the third in the world. This indicates that the influence of literature on land consolidation in China needs to be improved. Thailand leads the list, with an average of 57.75 citations per article. Niroula and Thapa [42], for example, analyzed the structural problems of land consolidation that did not address fragmentation, and concluded with an overview of a wide range of sustainable land consolidation policies and legal measures.

**Figure 8:**
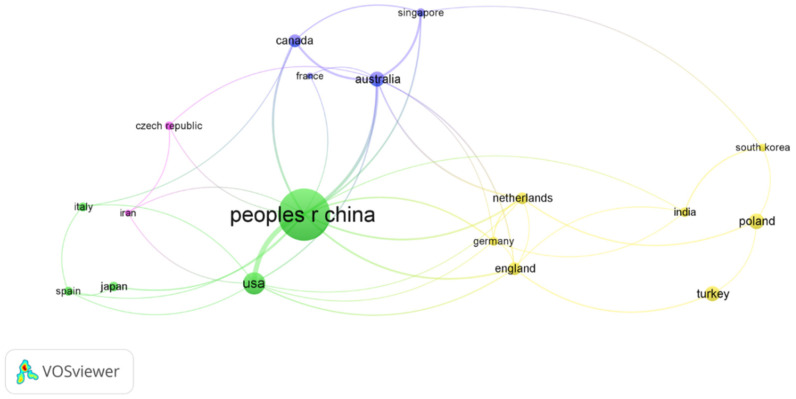
Country collaboration map in the field of land consolidation.

Based on the downloaded studies from the Web of Science database, we used VOSviewer software to screen out the cooperation between 18 countries (Figure 8). Country was set as the analysis unit, full counting was set as the counting method, and the minimum number of documents in each country was set to 7. The size of the circle in the figure indicates the number of papers published by each country. The finer the line between the two labels, the weaker the cooperation intensity between countries, and vice versa.

**Figure 9:**
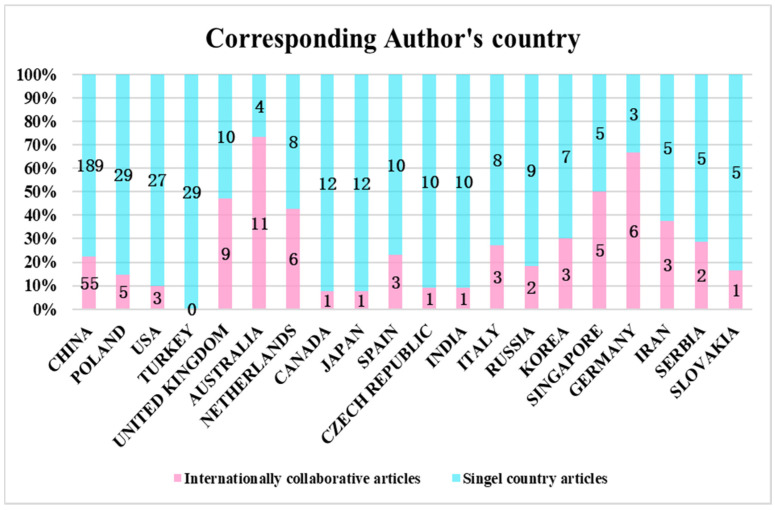
Corresponding authors’ nationalities in the 20 most prolific countries in the field of land consolidation.

As can be seen from Figure 8, China is at the center of international cooperation, followed by the United States, Australia, and Canada, with 31, 11, and 6 respectively. In total, 244 Chinese documents were sourced from state cooperation, accounting for about 22% of all published papers in China (Figure 9). China’s main cooperative countries were Australia and Germany, accounting for 73% and 66%, respectively. Among them, Australia and China, Singapore, and Canada constituted a cooperative, with frequencies of 11, 6, and 6, respectively. Germany published a total of nine documents in the field of land consolidation, mainly in cooperation with China and the Netherlands, with frequencies of three and two, respectively. In particular, the 29 papers published in Turkey were all independent research. The above indicates that international cooperation should be strengthened in the field of land consolidation.

### 3.5. Analysis of Keywords

#### 3.5.1. Analysis of High-Frequency Keywords

**Figure 10:**
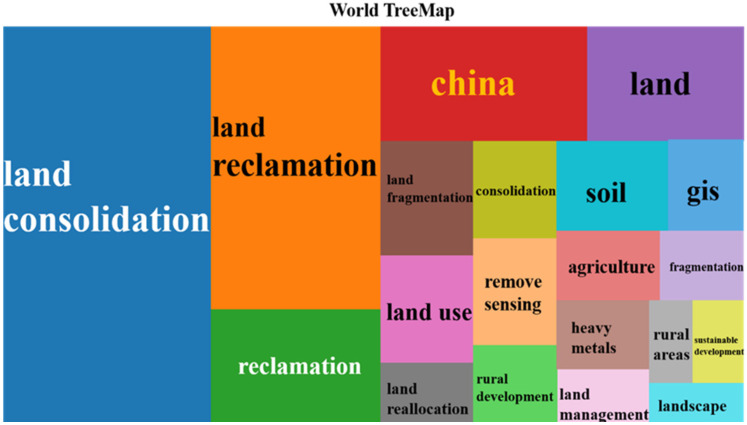
Word treemap of high-frequency keywords in the field of land consolidation.

Keywords are the high generalization of a research topic and content. Analysis of high-frequency keywords reflects the hotspots in the field of land consolidation in a straightforward way. The Bibliometrix and Biblioshiny installation packages in the *R* tool were adopted to count the author keywords and draw a Word TreeMap of the top 20 keywords in this area. Figure 10 shows that land consolidation, land reclamation, and reclamation accounted for over half of the total keywords, with proportions of 27%, 19%, and 9% respectively. The keyword “China” in the bright red box of Figure 10 accounted for 8% of the keywords, indicating that China was one of the main study areas of land consolidation studies. It corresponded to the total paper number and the state of international cooperation in China analyzed above. Remote sensing and GIS accounted for 2% of the keywords. This indicates that they are two major study tools in this area. For example, Karan et al. [44] used remote sensing and GIS to monitor the land degradation and reclamation of coal mines through the pioneering combination of ratio vegetation index (RVI), enhanced vegetation index (EVI), normalized vegetation index (NDVI), and normalized moisture index (NDMI).

Land fragmentation and fragmentation were the major problems to be solved by land consolidation, accounting for 4% and 2% respectively. Land fragmentation limits agricultural production and development in many countries, so it is urgent to address the problem of land fragmentation [45]. For example, Liu et al. [46] took Jiangsu Province of China as an example to analyze the distribution characteristics, influencing factors, and classification of arable land, and finally suggested that land fragmentation should be incorporated into the land consolidation plan so as to achieve high-quality and sustainable cultivated land development.

Finally, the proportions of land, land use, land reallocation, rural development, and sustainable development were lower, accounting for 1−2%. Even so, they have become an integral part of land consolidation policy, as there is a strong correlation between rural development and sustainable development and the issue of land fragmentation.

#### 3.5.2. Cluster Analysis and Multiple Correspondence Analysis of High-Frequency Keywords

**Figure 11:**
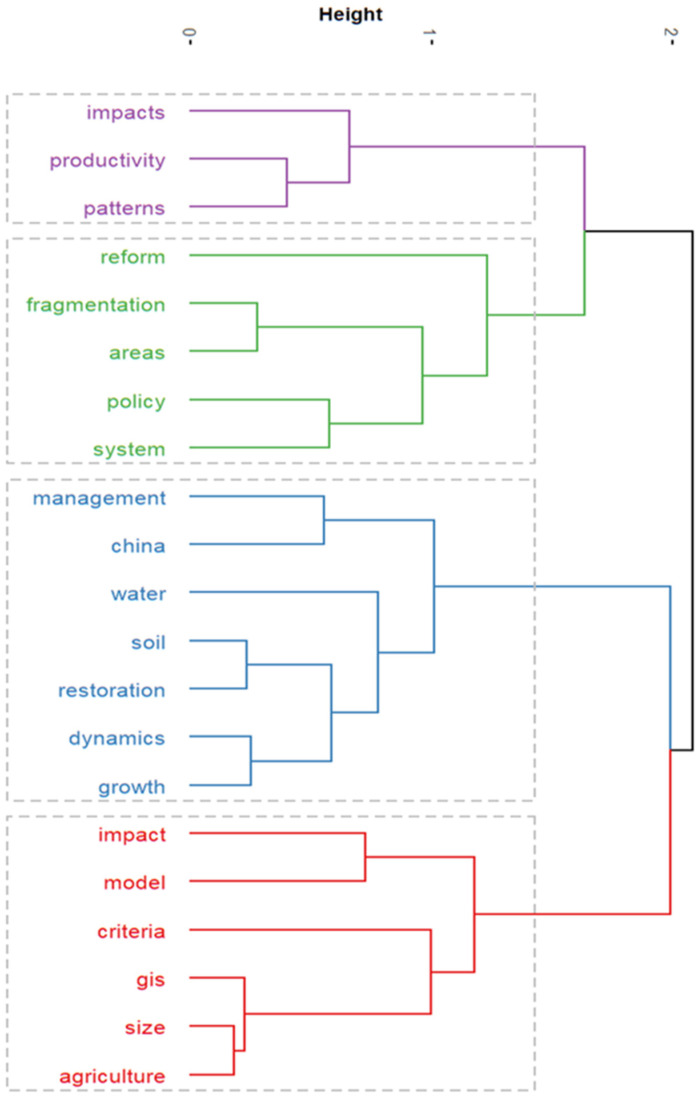
Tree dendrogram of hierarchical cluster analysis of keywords in the field of land consolidation.

Clustering analysis in literature metrology, based on the frequency of two or two key words appearing at the same time, uses statistical methods to simplify the complex keyword mesh relationship into a few relatively small groups of classes [47]. It is designed to detect the natural division of network groupings (clusters) based on similarity and to minimize similarity between clusters [48]. This study used hierarchical clustering to treat each clustered keyword as a category, then merged it among the clusters with the highest degree of similarity, and finally grouped all of the individuals into one category and demonstrated the similarity of the key words in the field of land consolidation research in the form of treemaps (Figure 11).

**Figure 12:**
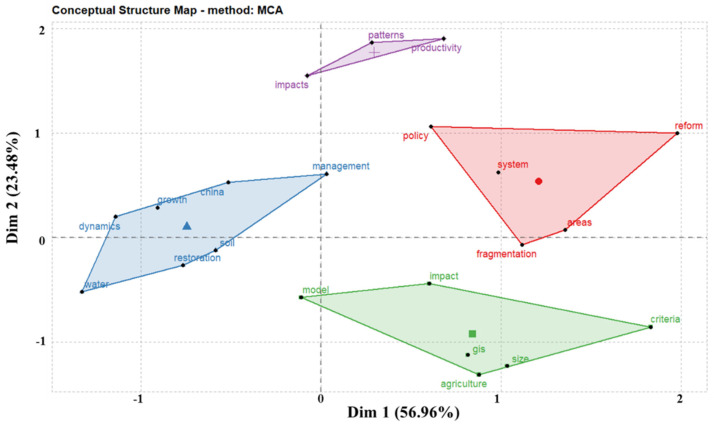
Multiple correspondence analysis of high-frequency keywords in the field of land consolidation.

Correspondence analysis first appeared in the 1960s, and has had a long and varied history [49,50]. It represents an exploratory approach of graphically representing the associations between variables in large, classified data sets to explore their relationships [51]. The corresponding analysis is designed to reveal the correspondence between different variables or categories of the same variable in qualitative data by lowering the dimension. Figure 11 and Figure 12 show the clustering results of multiple corresponding analyses in the field of land consolidation. It can be classified into four categories.

(1) The first category of cluster analysis: this category is mainly related to crop productivity, technical efficiency, arable land patterns, and landscape types. For example, Zeng et al. [52] used random cutting-edge analytical methods to calculate the efficiency of agricultural techniques for land consolidation. The results showed that the overall agricultural technology efficiency of producers was greatly improved after land consolidation, with an average technical efficiency of 0.924. Using the revised ecological connectivity index, the study of Wang et al. [53] in Da’an City, Jilin Province of China during 2008–2014 verified the negative impacts of land consolidation on ecosystem services, reflecting the problems in the implementation of the land consolidation project.
(2) The second category of cluster analysis: this category is mainly related to the impacts of land consolidation policies, reform, and land fragmentation. The review and prospects of land consolidation development, as well as the study of the influencing factors of land fragmentation, help the government to formulate relevant land consolidation policies. Based on the regional data of Jiangsu Province, Liu et al. [46] explored the characteristics, influencing factors, and classification of the spatial distribution of cultivated land, which is of great significance to the improvement of agricultural production capacity on the regional scale. At the same time, the fragmentation degree of cultivated land in the construction area is higher than that outside the construction area. The awareness of this situation helps the government to formulate relevant land consolidation policies.
(3) The third category of cluster analysis: based on population growth, this category mainly studies the impacts of land consolidation on ecosystem services through land-use change under the background of accelerated urbanization. It is represented by studies in China. For instance, using GIS-RS technology, Hao et al. [54] selected typical farmlands to analyze the change in the cropland ecosystem service value with land-use change in northeast China.
(4) The fourth category of cluster analysis: this category is mainly related to the benefit evaluation of land consolidation and the adoption of models. The benefit evaluation of land consolidation includes three aspects: economic benefit, social benefit, and ecological benefit. Based on land-use patch data, Shi et al. [55] combined landscape pattern analysis with production, life, and benefit evaluation to overcome the shortcomings of previous single-benefit evaluation and conduct comprehensive research on land consolidation projects. The results showed that land consolidation directly or indirectly improved the landscape ecological pattern of the project area, and land consolidation obviously improved the balanced distribution of cultivated land and the centralized distribution of construction land in the project area.

### 3.6. Evolution Analysis of Themes in the Field of Land Consolidation

According to Weismayer and Pezenka [56], it is important to study research development in a field in terms of themes and thematic evolution. Sankey diagrams, or Sankey energy shunts, are also known as Sankey energy shunts. Sankey diagrams describe the flow of different nodes in a network, and they are most typically used to analyze the flow of energy or matter. Arrows or direction lines are used to represent these flows, and the thickness of the arrows or direction lines is proportional to the flow size. These diagrams are commonly used in industrial ecology to describe product life cycle assessments and for rapidly visualizing energy efficiency in engineering [57]. Sankey diagrams emphasize the size and direction of traffic within the system, which, due to their wide practicality, have been applied to many geographic or human environmental research environments. Based on the Sankey diagram’s visual presentation of the changes in the subject matter of the land consolidation research field over time, this study can see the diversion of different topics in the field of land consolidation, and clarify the quantitative information, such as subject flow and conversion relationship [58].

**Figure 13:**
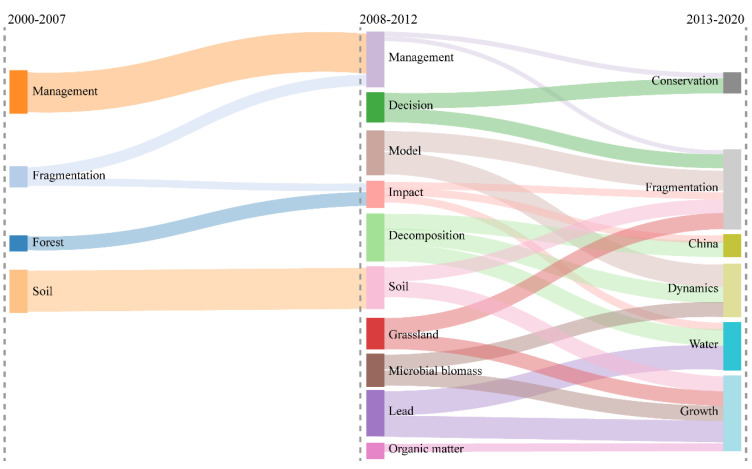
Thematic evolution of the land consolidation research field (2000–2020).

Referring to Zhou et al. [43], this paper divides the subject diversion of land consolidation development process into three stages, with 2007 and 2012 as breakpoints. Over the past 20 years, the research topics in the field of land consolidation have shown several evolutionary paths in three directions (Figure 13).

(1) Studies on land fragmentation related to land consolidation. ➀ Fragmentation→management→conservation, fragmentation. ➁ Fragmentation→impact→fragmentation, China, water. While enriching the structure and dispersing the risk of agricultural cultivation, and increasing farmers’ income, the fine fragmentation of cultivated land has also caused the waste of land resources and the increase of the agricultural production cost to a certain extent, thus reducing agricultural production efficiency and hindering the development of agricultural mechanization. Land consolidation is an effective way to reduce cultivated land fragmentation. For example, Ela [59] took Turkey as an example, and analyzed the impacts of land consolidation projects on agricultural land fragmentation. The proportions of agricultural enterprises with an index value of less than 0.40 were 1.17% and 3.7%, respectively, before land consolidation, and decreased to 0.6% and 2.3%, respectively, after land consolidation. The resulting values indicate a decrease in the degree of plot fragmentation in the area. Moreover, land consolidation projects have brought great economic benefits to the owners of agricultural enterprises in the region.
(2) Research on the development process of land consolidation. ➀ Management→management→conservation. ➁ Management→management→fragmentation. As time passed, the number of publications decreased significantly after 2012 with evolved themes. The development stages and priorities of land consolidation differed between countries. The study of the development process is conducive to the formulation of relevant policies. The focus of land consolidation in Germany has changed from adjusting farmland ecology and improving agricultural supporting facilities to the current stage of focusing on the transformation of rural infrastructure construction and regional planning, and the protection of natural landscapes; in addition, special attention is paid to public participation in land consolidation projects [60]. The focus of the current land consolidation work in the Netherlands is to enhance the comprehensive role of the land and strengthen the protection and improvement of the ecological environment in the process of renovation [61]. Land consolidation in Japan was initially aimed at the treatment of farmland salinization and the reclamation of land from the sea, but later developed into an overall plan of the region. Since the 1980s, land consolidation has been an important means to realize the symbiosis of residential space and agricultural land. The preparation of a comprehensive village development plan that maintains agricultural characteristics has fully demonstrated the important position and huge role of land consolidation in rural development [62]. Portugal believes that comprehensive land consolidation projects are geographically defined as rural land development activities [43].
(3) Research on the impacts of land consolidation on soil. ➀ Soil→soil→fragmentation, growth. ➁ Forest→impact→fragmentation, China, water. ➂ Microbial biomass→dynamics, growth. ➃ Decomposition→China, dynamics, water. ➄ Organic matter→growth. Soil is an important resource basis for agricultural production, as well as an important object of land consolidation. The direction of the thematic evolution is mainly between the effects of land consolidation behavior on soil physical structure, nutrient cycling, and microbial functions. This research is of great significance for avoiding the negative effects of land consolidation and improving the benefits of land consolidation. For example, He et al. [63] characterized the soil microbial communities under five land-use patterns, and used DNA fingerprinting and metabolic analysis as the characteristics, revealing that land reclamation has severely affected the population size, composition, and structure of soil microbial communities, as well as bacteria. In addition, Hou et al. [64] investigated soil samples of cinder-reclaimed land after reclamation periods of 1, 6, and 15 years, compared the characteristics of various soil microbial communities in the reclamation area, and compared areas not affected by coal mining. Soil sample analysis revealed that the application of microbial remediation technology can be used to adjust the structure of the soil microbial community, improve soil quality, and shorten the soil recovery cycle.

## 4. Conclusions and Discussion

Based on the Web of Science database to retrieve studies in the field of land consolidation during 2000–2020 and adopting the Bibliometrix and Biblioshiny software packages for data mining and analysis, the conclusions of this study are as follows.

(1) In view of year distribution and the number of publications, the development of land consolidation studies can be divided into three stages: 2000–2007 is the germination period, 2008–2012 is the growth period, and 2013–2020 is the high-yield period. The number of publications reached its peak in 2020, indicating that more and more attention was paid to this field.
(2) In terms of the distribution of research countries, the published papers were mainly from Asia and Europe. Among them, China had the largest number of publications, while Thailand had the greatest influence. The analysis of cooperation between countries showed that most studies were independent research, which was not conducive to the globalization of scientific research forces in land consolidation.
(3) The high-frequency keywords in land consolidation mainly included land consolidation, land reclamation, China, remote sensing, and land fragmentation. The future research directions included land consolidation, its impacts on ecosystem services, benefit evaluation of land consolidation, research combining land fragmentation, etc.
(4) The research on land consolidation presents three evolutionary paths, namely the study of land fragmentation related to land consolidation, the study of the developing process of land consolidation, and the study of the impacts of land consolidation on soil.

Based on existing literature, future studies on land consolidation can be carried out from the following aspects.

(1) Exploring new methods for benefit evaluation of land consolidation. At present, the evaluation method of land consolidation benefits is relatively single, and the standard for index selection is not uniform. The determination of index weight values is mostly qualitative. Future research should explore new benefit evaluation methods for land consolidation, improve the evaluation index system, and strengthen quantitative research on the benefit evaluation of land consolidation [65].
(2) The scale effect of the impacts of land consolidation on ecosystem services. Under the influence of land consolidation, the spatial heterogeneity and temporal dynamics of ecosystem services will change in time and space [66]. In view of the characteristics of land consolidation implementation and research, the coupling mechanism analysis of ecosystem services at different temporal–spatial scales and the construction of a multi-scale coupling model to achieve the integration of multi-scale land consolidation management and regulation is of important scientific significance and practical value.
(3) Research on the mechanism and comprehensive effects of land consolidation on soil. Future research is expected to focus on exploring the morphological characteristics, evolutionary process, and causative mechanism of the soil system under different remediation modes, types of areas, and remediation years [67]; continue an in-depth study of the impact of land consolidation on the overall soil environment; and establish a quantitative model of the impact of land consolidation on the soil environment.
(4) Multidisciplinary integrated system research on land consolidation. Land consolidation is a comprehensive system engineering involving natural resources, the economy, society, ecological environment, technology, etc. [68]. Current studies on land consolidation are mostly based on independent studies. It is necessary to strengthen the cooperation between experts and scholars in different fields and countries, which is of great significance for condensing innovative ideas, sharing research resources, and promoting the development of land consolidation.
(5) Research on land consolidation and rural revitalization. The Chinese Rural Revitalization strategy aims to establish a sound urban–rural integration development system, mechanism, and policy system, and accelerate the modernization of agriculture and rural areas. Based on this perspective, land consolidation should activate key development factors, such as the rural population, land, and industry, and coordinate the revitalization of material space and the promotion of a spiritual core. Under the unified spatial planning system, coordinate land consolidation planning and rural revitalization planning, and vigorously develop a new model that combines land consolidation and multi-functional agriculture [8].
(6) Strengthen theoretical research on land consolidation. Land consolidation has been practiced in many countries for many years, and there is an urgent need for theoretical improvement and systematic summary. It is necessary to make full use of the theoretical fruits of related disciplines to carry out solid internal theoretical research and promptly introduce innovative methods and research concepts from other disciplines so as to realize the continuous development of land consolidation [69].
(7) Promote 3S technology research and the application of land consolidation. Due to the complex process and high technical requirements, on-site investigation of land consolidation is difficult [70,71]. The time-consuming and laborious traditional survey methods perform poorly in locating, surveying, and recording tasks [72]. Applying 3S technology in land consolidation will help to solve these problems, and refers to RS, GIS, and GPS. Remote Sensing (RS) can be used to obtain various ground feature elements. The Global Positioning System (GPS) can be used for the spatial positioning of important features. The Geographic Information System (GIS) can be used for comprehensive processing and integrated management of land consolidation data [73]. As an efficient means of acquiring and managing spatial information, 3S technology has a broad application prospect in the field of land consolidation.
(8) Improve the supervision mechanism of land consolidation. A scientific and reasonable supervision and management mechanism is not only a guarantee for the smooth completion of land consolidation projects, but also has irreplaceable practical significance for promoting the harmonious development of localities [74]. It is essential to speed up the improvement of land consolidation supervision and management mechanisms, and implement joint responsibilities for land consolidation. Combine legal, administrative, technological, and other management methods to reasonably establish a government-led, land-based, departmental-collaborative, and public-participation working mechanism, effectively implement land consolidation objectives and responsibilities, and guarantee the completion quality and implementation level of land consolidation projects.

## Figures and Tables

**Table 1:** Top 10 local citation scores (LCS) in land consolidation research.

Documents	DOI	Year	LCS	GCS
TERRY VD, 2007, GEOFORUM	10.1016/j.geoforum.2006.11.010	2007	39	78
WU ZP, 2005, CHINA ECON REV	10.1016/j.chieco.2004.06.010	2005	17	81
CAY T, 2011, EXPERT SYST APPL	10.1016/j.eswa.2011.02.150	2011	16	30
YASLIOGLU E, 2009, EUR PLAN STUD	10.1080/09654310802553639	2009	14	20
SUZUKI T, 2003, MAR POLLUT BULL	10.1016/S0025-326X(02)00405-8	2003	12	48
ASLAN STA, 2007, SPAN J AGRIC RES		2007	11	21
ASIAMA KO, 2017, J RURAL STUD	10.1016/j.jrurstud.2017.09.007	2017	11	25
HOEKSEMA RJ, 2007, IRRIG DRAIN	10.1002/ird.340	2007	10	71
JIAO JJ, 2001, GROUND WATER	10.1111/j.1745-6584.2001.tb02479.x	2001	9	25
TOURINO J, 2003, INT J GEOGR INF SCI	10.1080/1365881031000072636	2003	9	26

**Table 2:** Top 10 global citation scores (GCS) in land consolidation research.

Documents	DOI	Year	LCS	GCS
YANG HY, 2011, BIRD CONSERV INT	10.1017/S0959270911000086	2011	6	90
WU ZP, 2005, CHINA ECON REV	10.1016/j.chieco.2004.06.010	2005	17	81
TERRY VD, 2007, GEOFORUM	10.1016/j.geoforum.2006.11.010	2007	39	78
HOEKSEMA RJ, 2007, IRRIG DRAIN	10.1002/ird.340	2007	10	71
SIKOR T, 2009, WORLD DEV	10.1016/j.worlddev.2008.08.013	2009	6	63
LIU SL, 2013, ECOL ENG	10.1016/j.ecoleng.2012.12.001	2013	9	54
SUZUKI T, 2003, MAR POLLUT BULL	10.1016/S0025-326X(02)00405-8	2003	12	48
ADRIANSEN HK, 2009, GEOFORUM	10.1016/j.geoforum.2009.05.006	2009	5	37
MUCHOVA Z, 2016, ECOL ENG	10.1016/j.ecoleng.2016.01.018	2016	8	31
CAY T, 2011, EXPERT SYST APPL	10.1016/j.eswa.2011.02.150	2011	16	30

**Table 3:** Top 10 influential authors in the field of land consolidation.

Author	h-Index	g-Index	m-Index	TC	NP	PY-Start
LIU Y	7	12	0.7	349	12	2012
ZHOU Y	7	12	1	197	12	2015
JIN X	7	11	1	161	11	2015
LI Y	7	10	0.875	455	10	2014
LEN P	7	9	1.167	114	9	2016
ARULRAJAH A	6	7	0.333	170	7	2004
BO MW	6	7	0.333	170	7	2004
DEMETRIOU D	6	6	0.6	180	6	2012
ZHANG Y	5	10	0.833	104	11	2016
CAY T	5	8	0.417	196	8	2010

## Data Availability

Not applicable.
